# Supplementary material for: Segmentation of dense and multi-species bacterial colonies using models trained on synthetic microscopy images
Source: PLoS Comput Biol. 2025 Apr 4;21(4):e1012874. doi: 10.1371/journal.pcbi.1012874 (PMC11970677; doi:10.1371/journal.pcbi.1012874)
Supplement: S1 Appendix — Detailed description of synthetic image generation and training procedure for segmentation models and cycleGAN. Also contains analysis of how accurately cell properties are measured using different segmentation models as a function of IoU. (PDF) [file pcbi.1012874.s001.pdf]

Supplemental Information: Segmentation of dense and multi-species bacterial colonies using models trained on synthetic microscopy images

Vincent Hickl,* Abid Khan, René M. Rossi,
Bruno F. B. Silva, and Katharina Maniura-Weber

* Email address; vincent.hickl@empa.ch

CONTENTS

1. Synthetic image generation S3
   A. Rod-shaped cells and cell alignment S3
   B. Circular cells S5
   C. Clustering S6

2. Segmentation Model Training Parameters S7

3. CycleGAN training S8

4. Segmentation accuracy as a function of intersection over union S10

References S13

1. SYNTHETIC IMAGE GENERATION

The raw (unprocessed) synthetic images of bacteria were binary images produced using custom Python functions. All code is publicly available as described in the main text.

In short, we begin by specifying the dimensions of the images, and generating an array filled with zeros that serves as a "canvas" on which to draw cells. Here, the canvas was chosen to be 2048×2048 pixels in size, but synthetic images are later split into smaller images for processing and training. Then, for each cell to be drawn, the program determines the set of pixels that should be set to one to draw cells of the desired shape, size, and orientation on the canvas. Before drawing each cell, the algorithm checks how much this set of pixels overlaps with other cells to avoid excessive overlap. Cells with overlap above a chosen threshold are not drawn. Additionally, an option may be chosen to draw cells only in clusters, in which case cells are only drawn if they are within a predefined distance of previously drawn cells. In generally, cell positions, dimensions, and orientations are chosen randomly from empirically determined distributions. Optionally, for rod-shaped cells, the orientation of each cell may instead be chosen to be the same as its nearest neighbor to simulate the alignment commonly observed in bacterial monolayers. In this case, a noise parameter is also introduced so the alignment is not exact, adding some fluctuations to the cell alignment. By combining these parameters and options in different ways, a diverse set of images can easily be produced to ensure the resulting segmentation algorithm is well-suited to study real experimental images.

Along with the binary image of bright cells on a dark background (which can be inverted to produce brightfield synthetic images), the algorithm produces a mask containing unique integer labels for each cell to be used for training of the segmentation algorithm.

Below, additional details of the methods for drawing each cell type and for the different methods within the algorithm are provided.

A. Rod-shaped cells and cell alignment

Pseudomonas aeruginosa are modeled as rectangles of width w and length l with hemispherical caps of radius $w/2$. Thus, the actual length of each cell is $l + w$. The orientation of the cell is given by the angle θ, measured with respect to the vertical, as shown in Fig A.

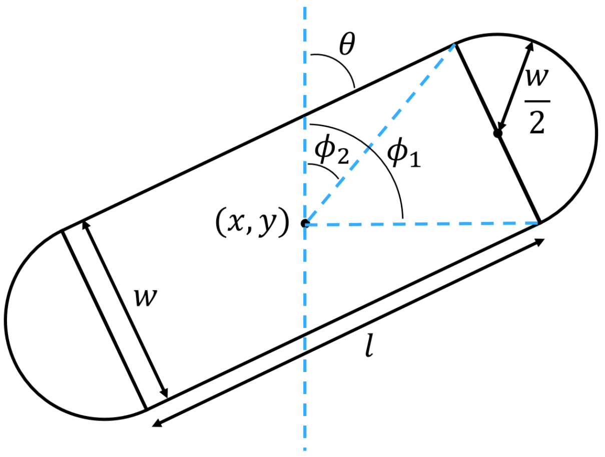

FIG. A. Diagram of model rod-shaped cell.

First, the coordinates of the vertices of the rectangle are determined:

$$c = \{x + d\sin\phi_1,\ x + d\sin\phi_2,\ x - d\sin\phi_1,\ x - d\sin\phi_2\} \tag{1}$$

$$r = \{y + d\cos\phi_1,\ y + d\cos\phi_2,\ y - d\cos\phi_1,\ y - d\cos\phi_2\} \tag{2}$$

where, c and r refer to the columns and rows of the image array, (x, y) is the center of the cell (its location), $d = \frac{1}{2}(w^2 + l^2)^{1/2}$ is half the size of the rectangle's diagonal, and the angles $\phi_1 = \theta + \tan^{-1}(w/l)$ and $\phi_2 = \theta - \tan^{-1}(w/l)$ are the angles between the center of the rectangle and the vertices. The endcaps are then simply drawn as circles with radius $w/2$ centered on the short sides of the rectangle. The `draw.polygon` and `draw.ellipse` from the `scikit-image` library in Python are then used to obtain the set of pixels for each cell.

The lengths and widths of the rods are randomly drawn from distributions determined empirically by manually measuring the dimensions of 30-50 randomly chosen cells in the real microscopy images to be analyzed. We found that cell widths follow a normal distribution with a mean of $\bar{w} = 0.8$ μm and a standard deviation of $\sigma_w = 0.1$ μm. Cell lengths follow a normal distribution with $\bar{l} = 2.7$ and $\sigma_l = 0.6$. In practice, we believe it is beneficial to increase the standard deviation slightly when generating synthetic images to ensure the segmentation algorithm can accuartely produce masks for a variety of real cells. Further testing is required to determine which parameters lead to optimal segmentation results.

Once the set of pixels corresponding to the cell is found, the algorithm checks how many of these pixels are already 1. The amount of overlap permitted between cells can be chosen via

the parameter `thresh`, which is the maximum number of overlapping pixels above which the new cell is not drawn. The default value chosen here is `thresh` $= 5$, but this depends heavily on the magnification, camera, and experimental conditions used to capture a particular image.

If the number of overlapping pixels exceeds the threshold, the algorithm automatically rotates the cell from θ to $\theta + \pi$ in increments of 0.1, checking at each step whether the cell can now be drawn. This process reduces the number of cells that are not drawn because of excessive overlap, and introduced a small amount of cell alignment even when orientations are initially chosen randomly. If the cell cannot be drawn after all orientations have been tried, it is simply discarded and the algorithm moves on to the next set of coordinates and dimensions. This approach of removing cells that don't fit in a space introduces a slight bias towards smaller cells, but our results do not suggest that this prevents the segmentation model from accurately detecting large cells, nor does it lead to oversegmentation of rod-shaped cells (see Fig 2 in the main text).

To simulate the tendency of rod-shaped cells to align, an option may be chosen to set each cell's orientation to $\theta_j + \epsilon$, where θ_j is the orientation of the cell's nearest neighbor and ϵ is a small random noise parameter to introduce fluctuations in the alignment. In this work ϵ is chosen from a normal distribution with mean 0 and standard deviation 0.1 radians. The standard deviation can be set to vary the degree of cell alignment across different images. To generate images of aligned cells, a small number (usually 10-20) of randomly positioned and oriented cells is first generated, before the remainder of the cells is generated to be aligned with their neighbors. To efficiently find the cells' nearest neighbors, a K-D tree was created from the set of cell coordinates using the existing methods in the SciPy library for Python. This K-D tree is a data structure which can be queried to efficiently find nearest neighbors and distances between cell pairs.

B. Circular cells

Staphylococcus aureus are modeled as circles of radius r. Their positions and dimensions are randomly chosen as for the rod-shaped cells above. Cell radii were manually measured and found to follow a normal distribution with a mean radius of $\bar{r} = 0.65$ µm and a standard deviation of $\sigma_r = 0.10$. It is common for *S. aureus* to remain tightly clustered after dividing,

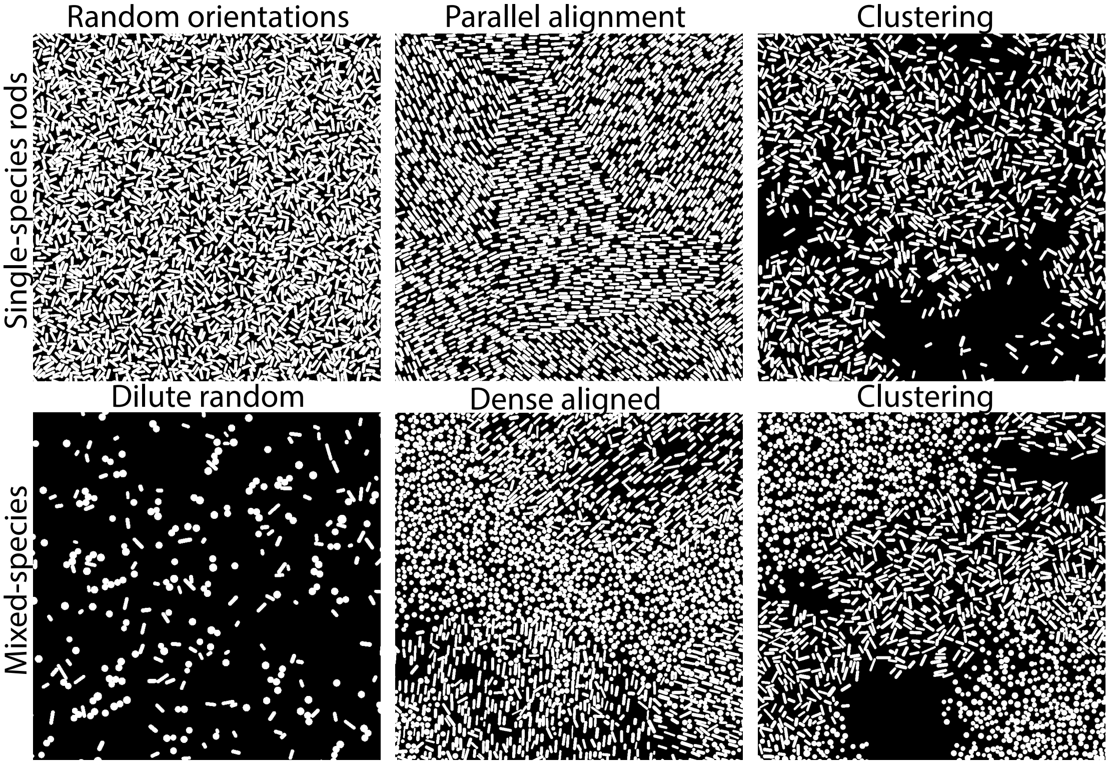

FIG. B. Sample raw synthetic images, showing different arrangements of cells produces via different input parameters.

so to mimick this behavior and produce more realistic synthetic images a higher overlap threshold of 15 was chosen compared to the rod-shaped cells.

C. Clustering

For both cell types, it is possible with our algorithm to produce images where cells tend to cluster together, meaning there are distinct regions of densely packed cells and more dilute regions, and the two cell types tend to be more segregated. When this option of 'clustering' is chosen, cells are only drawn if they are within a certain distance $r_{cluster}$ of the nearest neighbor of the same species. Similar to the option of aligning rod-shaped cells, this clustering method only works after some cells are already present, so the creation of these images is performed in multiple steps. As before, a K-D tree is created for each species to determine nearest neighbor distances.

Together, these methods enable the creation of diverse synthetic images with rod-shaped and circular cells that are either clustered or randomly distributed and, in the case of

rods, randomly oriented or aligned to varying degrees. A set of sample images created with different input parameters is shown in Fig B. These images thuse provide excellent training data for segmentation models suitable to different systems and imaging techniques. The extension of this approach to objects with other shapes only requires the creation of functions to draw those shapes.

2. SEGMENTATION MODEL TRAINING PARAMETERS

Within the Omnipose command line interface (CLI), the training command used to train the model for segmentation of monolayers of *Pseudomonas aeruginosa* (*P. a.*) was

```
omnipose --train --use_gpu --dir <image_folder_path> --mask_filter
_masks --n_epochs 2000 --pretrained_model None --learning_rate 0.1
--diameter 0 --batch_size 16 --RAdam --omni --nclasses 3 --tyx 224,224
--save_every 100 --save_each --verbose
```

In general, it is not guaranteed that the model trained at the last epoch produces the best segmentation results, so the model was saved at regular intervals to evaluate performance.

For all models trained to segment bacteria in mixed colonies of *P. a.* and *S. a.* (either in confocal or brightfield microscopy images), the training command used was

```
omnipose --train --use_gpu --dir <image_folder_path> --mask_filter
_masks --n_epochs 2000 --pretrained_model None --learning_rate 0.1
--diameter 0 --batch_size 16 --RAdam --omni --nclasses 3 --tyx 128,128
--save_every 100 --save_each --verbose
```

In all cases, the training converged quickly enough that no significant improvements could be obtained after 2000 epochs, and in some cases the best performance was obtained for models from earlier epochs. The difference in the parameter `--tyx` between the two commands is due to the difference in the size of the images in the training sets, as described in the main text.

3. CYCLEGAN TRAINING

As done in Ref. [1], we use discriminators for the microscopy images as well as for their Fourier transforms. For the real-space discriminators $D_{X,\text{img}}$, and $D_{Y,\text{img}}$, the loss functions are given with respect to the generators $G : X \to Y$ and $F : Y \to X$:

$$\mathcal{L}\left[D_{X,\text{img}}\right] = \frac{1}{|\mathcal{D}_y|}\sum_{y\in\mathcal{D}_y}\|D_{X,\text{img}}(F(y))\|_2 + \frac{1}{|\mathcal{D}_x|}\sum_{x\in\mathcal{D}_x}\|1 - D_{X,\text{img}}(x)\|_2, \tag{3}$$

$$\mathcal{L}\left[D_{Y,\text{img}}\right] = \frac{1}{|\mathcal{D}_x|}\sum_{x\in\mathcal{D}_x}\|D_{Y,\text{img}}(G(x))\|_2 + \frac{1}{|\mathcal{D}_y|}\sum_{y\in\mathcal{D}_y}\|1 - D_{Y,\text{img}}(y)\|_2 \tag{4}$$

and similarly for Fourier-space discriminators $D_{X,\text{FFT}}$, and $D_{Y,\text{FFT}}$. Here $\mathcal{D}_x$ and $\mathcal{D}_y$ are the datasets for the real and simulated microscopy images, respectively, and $\|\cdot\|_2$ is the L2 norm operator. For the generators, the loss functions are

$$\mathcal{L}\left[G\right] = \mathcal{L}_{\text{adv}}[G] + \lambda_{\text{cyc}}\mathcal{L}_{\text{cyc}}[F,G] + \lambda_{\text{id}}\mathcal{L}_{\text{id}}[G] \tag{5}$$

$$\mathcal{L}[F] = \mathcal{L}_{\text{adv}}[F] + \lambda_{\text{cyc}}\mathcal{L}_{\text{cyc}}[F,G] + \lambda_{\text{id}}\mathcal{L}_{\text{id}}[F] \tag{6}$$

Here, the adversarial loss $\mathcal{L}_{\text{adv}}$ is the term that aims to fool its respective discriminator(s):

$$\mathcal{L}_{\text{adv}}[G] = \frac{1}{|\mathcal{D}_x|}\sum_{x\in\mathcal{D}_x}\|1 - D_{Y,\text{img}}(G(x))\|_2 + \frac{1}{|\mathcal{D}_x|}\sum_{x\in\mathcal{D}_x}\|1 - D_{Y,\text{FFT}}(G(x))\|_2 \tag{7}$$

The cycle-consistency loss is given by

$$\mathcal{L}_{\text{cyc}}(F,G) = \frac{1}{|\mathcal{D}_x|}\sum_{x\in\mathcal{D}_x}\|F(G(x)) - x\|_1 + \frac{1}{|\mathcal{D}_y|}\sum_{y\in\mathcal{D}_y}\|G(F(y)) - y\|_1 \tag{8}$$

and the identity loss is given by

$$\mathcal{L}_{\text{id}}(G) = \frac{1}{|\mathcal{D}_y|}\sum_{y\in\mathcal{D}_y}\|G(y) - y\|_2. \tag{9}$$

For the cycle-consistency loss, λ_{cyc} was 10, and for the identity loss, λ_{id} was 5. Fig C shows the loss functions of the discriminators and generators as a function of the epoch in training. All the models are constructed with Tensorflow and run locally. There, training a c ycleGAN model from scratch takes around 8 hours to complete using a single NVIDIA 3090 GPU.

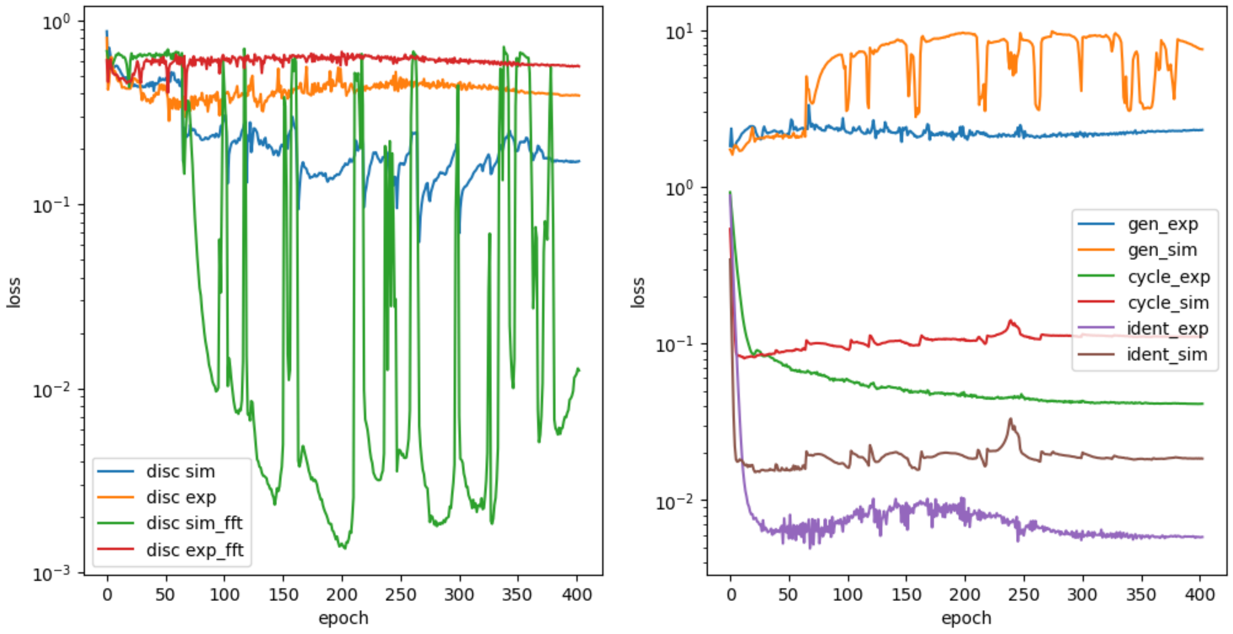

FIG. C. Losses of the discriminators (left) and the generators (right) vs training epoch.

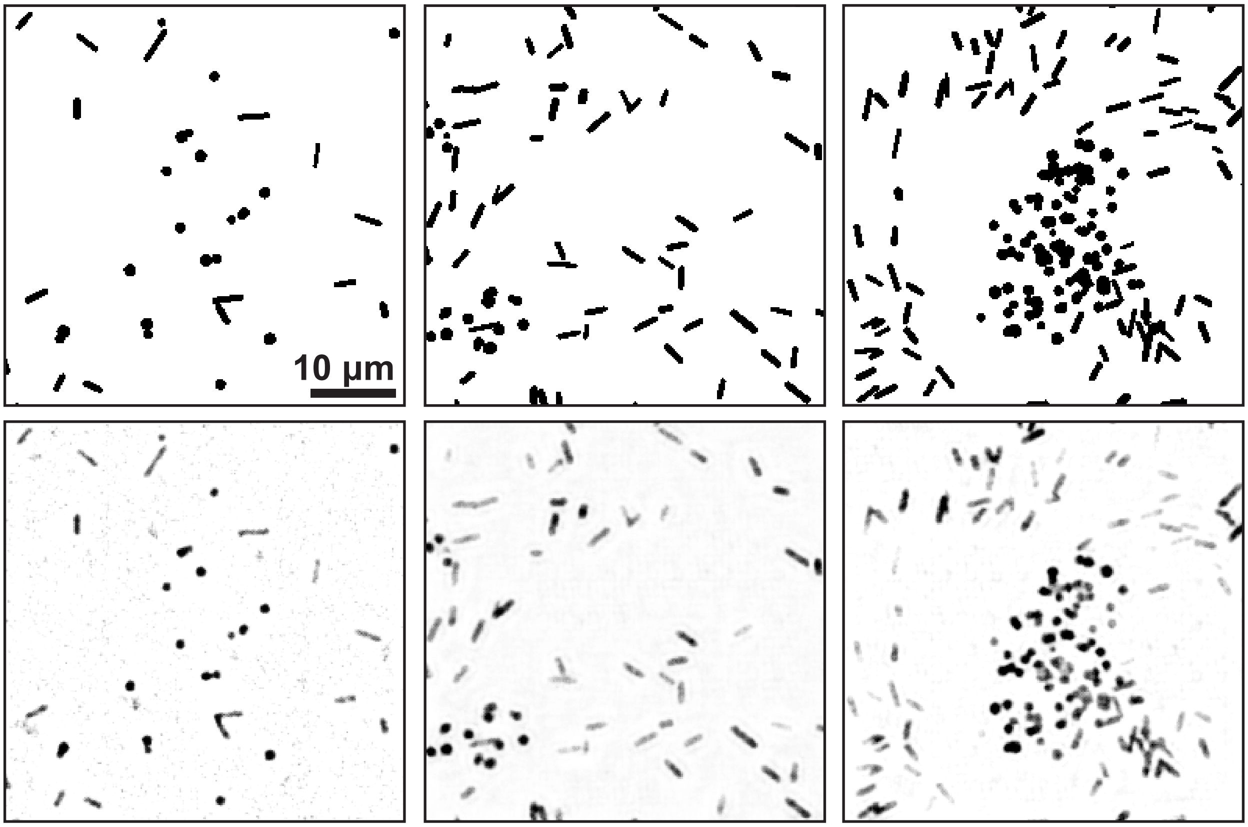

FIG. D. Synthetic brightfield images before (top) and after (bottom) processing by a cycleGAN.

4. SEGMENTATION ACCURACY AS A FUNCTION OF INTERSECTION OVER UNION

To quantify the ability of the segmentation models to provide accurate measurements of cell properties, differences between ground truth masks and masks produced by the segmentation models were analyzed as a function of the intersection over union (IoU). This analysis was performed for the model trained on synthetic images processed by a cycleGAN for dense monolayers of *Pseudomonas aeruginosa* ("Synthetic model"), the model trained on synthetic images produced by SyMBac ('SyMBac model') [2], and on the pre-trained model trained on hand-annotated real images ("Bact_fluor_omni") [3]. The images analyzed were the same as those used to obtain the results of Fig 3, and the ground truth masks were obtained through hand-annotation. It should be noted that, because imaging through the sheet of PDMS produced images with a low signal-to-noise ratio, hand-drawn masks are not guaranteed to correspond perfectly to the 'real' cells. For very densely-packed regions especially, individual cell boundaries can be hard to identify, meaning differences between the hand-drawn labels and the labels from the segmentation models may not necessarily be errors, but rather the result of a different attempt to divide the bright regions in the image into discrete cells. For each cell in the hand-drawn ('ground truth') masks and its corresponding candidates from the two segmentation models, several key properties are measured: the cell position, orientation, aspect ratio, and total area. These properties are important for further analysis of the bacterial colony, particularly self-organization as described in Fig 4. Differences or errors between the ground truth values of these properties and the values from the segmentation models are then calculated. Additionally, the IoU between each candidate and the ground truth cell is calculated to determine appropriate IoU cutoffs to count true positives (TPs).

The synthetic model trained on images processed by a cycleGAN, the model from SyMBac, and the model pretrained on hand-annotated experimental images all accurately measure cell positions when the IoU is large. For both models, distances between the centroid of the ground truth cell and the candidate cell can be large when the IoU is small, as shown in Fig EA. Recall that the synthetic model typically produces cell labels with higher IoU than Bact_fluor_omni and the SyMBac model, meaning these errors are more common with these models, especially Bact_fluor_omni. Here, a natural choice for what can be considered

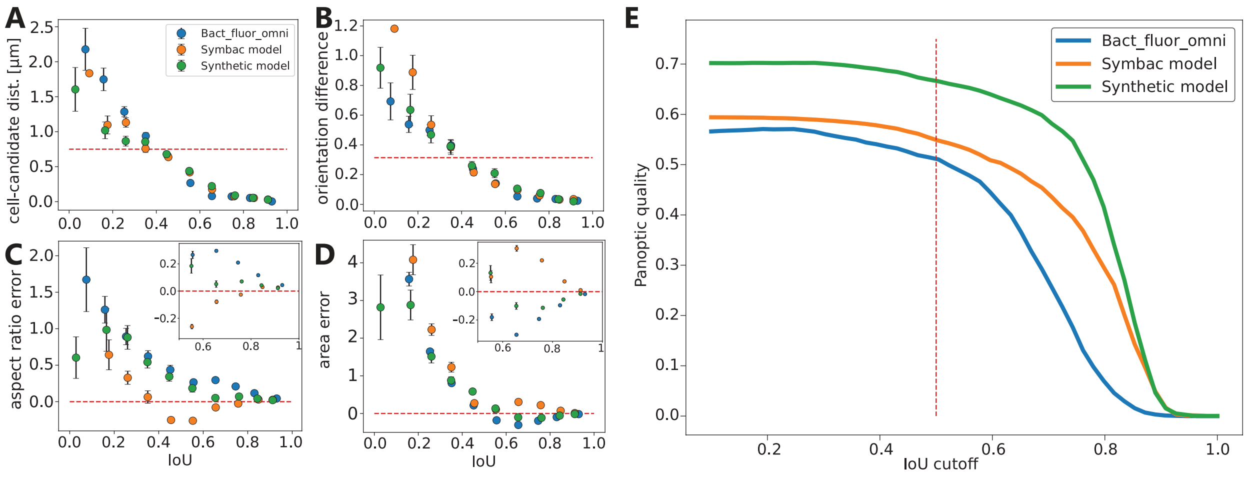

FIG. E. Analysis of model accuracy as a function of intersection over union (IoU). **a** Distance between ground truth cells and candidates produced by the segmentation models as a function of IoU. Red line represents mean cell width (0.75 µm). **b** Absolute value of difference in orientations between cells and candidates as a function of IoU. Red line represents $\pi/10$ radians. **c** Error in aspect ratio measurement from candidates compared to ground truth. Inset: Closeup of data for $IoU > 0.5$. Red line at 0. **d** Error in area measurement from candidates compared to ground truth. Inset: Closeup of data for $IoU > 0.5$. Red line at 0. **e** Panoptic quality as a function of IoU cutoff for counting true positives. Red line shows IoU cutoff of 0.5 used throughout this work.

an 'accurate' determination of the cell position is any case where the cell-candidate distance is less than the mean cell width, or about 0.75 µm (red dashed line). Cells from all three segmentation models meet this criterion, on average, when $IoU \gtrsim 0.4$. For the threshold of $IoU > 0.5$ chosen for this study, the distance is less than 0.5 µm, on average.

All three models accurately determine cell orientations for large IoU. Differences in cell orientations (reported here as absolute values) between ground truth cells and corresponding candidates exhibit a sharp decrease with IoU at low IoU, and level off at high IoU, as shown in Fig E. Note that a difference of $\pi/2 \approx 1.57$ corresponds to the case where the candidate cell is perpendicular to the ground truth. Here, a threshold of $\pi/10$ is chosen for what is considered an accurate measurement (red dashed line). Again, the three models accurately measure orientations, on average, when $IoU \gtrsim 0.4$. For the chosen threshold of $IoU > 0.5$, the difference in angle is typically less than 0.2 radians.

The two models trained on synthetic images provides more accurate measurements of cell

aspect ratios and areas than the model trained on experimental images. For the aspect ratio η, the error in the measurement is defined as $\eta_c/\eta_g - 1$, where η_g is the aspect ratio of the ground truth cell and η_c is the aspect ratio of the candidate. Similarly, the error for the area A is defined as $A_c/A_g - 1$. For all models, these errors can be quite large for low IoU, as shown in Fig Ec and Ed. Above the threshold of $IoU > 0.5$, the mean error for candidates produced by the synthetic model is less than 20%, whereas the error for candidate cells from the other two (especially Bact_fluor_omni), sometimes remain higher than 20%. In the range $0.6 < IoU < 0.9$, the synthetic model measures aspect ratios more accurately than the model trained on real images, and measures cell areas better than both the other models, as shown in the insets of Fig Ec and Ed. The synthetic model and Bact_fluor_omni tend to overestimate the aspect ratio and underestimate the cell area, on average, whereas the opposite is generally true of the SyMBac model.

Overall, these results show that, for $IoU > 0.5$, the model trained on synthetic images processed by a cycleGAN produces segmentation masks than can be used to accurately measure these cell properties and quantify bacterial self-organization. Thus, the choice of $IoU > 0.5$ as a threshold for true positives is justified for this study. However, for a detailed analysis of cell morphology where lower errors a required, only candidates with very high IoU (e.g. $IoU > 0.8$) may be sufficient. With such a high threshold, the measured quality of both segmentation models decreases significantly.

To quantify the overall quality of the segmentation models as a function of IoU cutoff, the panoptic quality (PQ, defined in the main text), is calculated for different IoU cutoffs, as shown in Fig Ee. For the synthetic model, PQ drops off sharply above an IoU cutoff of about 0.7. For Bact_fluor_omni and the SyMBac model, this drop-off begins at cutoffs of about 0.55 and 0.65, respectively. For all values of the IoU cutoff, PQ is significantly larger for the synthetic model. This result shows that, if highly accurate segmentation masks are required, both models should be used with caution, but the synthetic one provides better accuracy for the noisy images acquired in this work.

Importantly, the SyMBac model was trained on synthetic images not specifically created to resemble those obtained in the experiments carried out here, which have a lower signal-to-noise ratio and larger point-spread-functions. It is possible that SyMBac could be used to create synthetic images which closely resemble these images, in which case a model trained on them would perform more similarly to the model trained on synthetic images

processed by cycleGAN. However, the process of creating synthetic images with SyMBac requires significantly more effort than the method described here. The unique ease with which realistic synthetic images can be created using cycleGANs is a crucial advantage of our approach, making it ideally suited for the creation of bespoke segmentation models for different experimental conditions.

---

[1] Abid Khan, Chia Hao Lee, Pinshane Y. Huang, and Bryan K. Clark. Leveraging generative adversarial networks to create realistic scanning transmission electron microscopy images. *npj Computational Materials*, 9(1):1–9, May 2023. ISSN 2057-3960. doi:10.1038/s41524-023-01042-3. URL https://www.nature.com/articles/s41524-023-01042-3.

[2] Georgeos Hardo, Ruizhe Li, and Somenath Bakshi. Quantitative microbiology with widefield microscopy: navigating optical artefacts for accurate interpretations. *npj Imaging 2024 2:1*, 2(1):1–16, sep 2024. ISSN 2948-197X. doi:10.1038/s44303-024-00024-4. URL https://www.nature.com/articles/s44303-024-00024-4.

[3] Kevin J. Cutler, Carsen Stringer, Teresa W. Lo, Luca Rappez, Nicholas Stroustrup, S. Brook Peterson, Paul A. Wiggins, and Joseph D. Mougous. Omnipose: a high-precision morphology-independent solution for bacterial cell segmentation. *Nature Methods*, 19(11):1438–1448, October 2022. ISSN 1548-7105. doi:10.1038/s41592-022-01639-4. URL https://www.nature.com/articles/s41592-022-01639-4.
