# Supplementary material for: Segmentation of dense and multi-species bacterial colonies using models trained on synthetic microscopy images
Source: PLoS Comput Biol. 2025 Apr 4;21(4):e1012874. doi: 10.1371/journal.pcbi.1012874 (PMC11970677; doi:10.1371/journal.pcbi.1012874)
Supplement: Attachment — Submitted filename: PCOMPBIOL-D-24-01793 Review.pdf [file pcbi.1012874.s002.pdf]

In this manuscript, the authors present an exciting and novel method for training a cell segmentation model: simulating cell geometries to create artificial masks, applying an image-to-image translation using a cycleGAN to generate synthetic images similar to real images, and then training image segmentation models which have only used synthetic images and masks as ground truth data. Previous methods to generate synthetic images have involved complex simulations of cell growth and imaging conditions; the simulations for the current method are simply based on cell morphology, and the method is amongst the first of its kind for bacterial cells.

This method is highly significant because i) the use of synthetic images and annotation as ground truth data solves the issue of time-consuming human annotation as a major bottleneck of model training, ii) there is no bias in annotation, and iii) synthetic images can be adjusted to accommodate various imaging and sample conditions, amongst other advantages.

In its current state, I feel that the manuscript presents more of a proof of concept of the method, which will be highly adaptable for various experiments. An experimentalist would be interested in using the trained models, which are not made available. Thus, I believe it is important for the language of the manuscript to be more precise.

I have a few big picture critiques:

1. It would be helpful to make a stronger language distinction between “masks”, “raw synthetic images”, “synthetic images” and “processed synthetic images” within the manuscript. For example, l. 52 uses “synthetic microscopy images” when I believe this should be referring to masks. L. 167 refers to synthetic images, but I believe these would be the processed synthetic images. I think l. 275 is referring to processed synthetic images. This language change would make it easier for readers to understand and recreate the process.
2. Throughout the paper, an IoU>0.5 is used. However, this is very low, as IoU>0.8 is highly preferred for accurate predictions (see Avoiding a replication crisis in deep-learning-based bioimage analysis - Laine 2021).
3. Results: The meaning of the IoU metric in the context of this paper is ambiguous. As IoU is found from comparing results on a pixel level, images of cells with noisy or poorly defined boundaries could result in very different IoU values. Furthermore, since the Omnipose fluorescence model was trained with cytoplasmic and membrane fluorescence while the synthetic model is trained and tested on nucleic acid stains, should the performances be able to be compared?
4. The section headers in the Results section could be stronger. For example, “Segmenting dense *P. aeruginosa* monolayers” could mention outperforming existing models.
5. The resulting masks of the multi-species segmentation are very poor (Figure 5)--for example, some of the spherical or rod-shaped cell masks have sharp points. Is this a

result of poor image quality, lacking training data for the model, or another factor; how could these results be improved?

6. The multi-species capability seems to depend on the model being trained to recognize/ignore different cell morphologies. Would the authors expect this method to be able to differentiate between different species with similar morphologies (ex, *P.a.* and *E. coli* in the same image, both rod-shaped)?

MINOR COMMENTS:

1. The method is mentioned to work for both confocal and brightfield microscopy, however, the manuscript focuses heavily on the results for confocal microscopy. The authors support the idea of only using a single fluorescence channel/stain and using cell morphology to distinguish cells. As brightfield images are subject to less noise and photobleaching, in addition to having better defined boundaries, I would have thought that demonstrating the method for brightfield imaging would make a stronger case. Furthermore, the method was not shown for dense brightfield images, where cell contact would present a larger issue as compared to SYTO 9 stains, which are less likely to have overlap. I would also like to see an example of the processed synthetic brightfield images.
2. Verb tense in Author summary: We apply… and found…
3. Author summary: "multiple imaging methods, including both brightfield and confocal microscopy" - were other imaging methods investigated?
4. May add this reference regarding synthetic images to l. 33 https://www.nature.com/articles/s44303-024-00024-4
5. L. 134: wass -> was
6. L. 147: compared it to -> compared to
7. L. 147: Suggest rewriting the sentence for clarity, such as: The FID between the real dataset and the processed synthetic dataset was used to quantitatively compare sets of synthetic images to real images.
8. L. 152: generated by the cycleGAN… using the Omnipose package. (add the, remove for Python)
9. L. 173: "false negative positive rate" is slightly confusing.
10. The language in l. 187-191 could be more precise.
11. L. 214: Could be more clear with the term "associated". Is the synthetic image directly generated from the mask/the mask generated from the synthetic image?
12. L. 250: Don't see clusters of cells in Fig2 bottom right.
13. Fig 3 caption: "intersection over union (a common measure of how accurately cells are identified during segmentation)" This language could be more precise. IoU measures accuracy on a pixel-level rather than just measuring if cells are or are not identified.
14. Fig 4: Color is very important in this image, however, some cells have pixels of multiple colors.
15. Fig 4 caption: analyzed here include images -> analyzed here are extracted from images
16. L. 294: could mention undersegmentation
17. L. 313: models like it -> similar models

18. L. 327: provided -> provides
19. L. 351: decrease -> decreases
20. Section “Single-cell statistics from dense *Pseudomonas* monolayers”: This section could benefit from a structure more like “we expect… we find/show…” It was ambiguous whether the first line of each paragraph was a conclusion from the data or an expected relationship.
21. L. 419: possibly missing the word ‘high’ before “cell densities”?
22. L. 436-444: I found this section confusing. Wasn’t the model trained to identify circular cells for *S.a.*, while the model to identify non-circular (rod-shaped?) cells for *P.a.*? The *P.a.* model is also referred to as the *P. aeruginosa* model. It may be helpful to have consistent names for these models.
23. Citations: bacterial names should be italicized for ref.s 6, 9, 25, 26, 27, 29, 34
24. Supplemental Material: c ycleGAN -> cycleGAN
25. Perhaps beyond the scope of this manuscript, but it may be interesting to make a comment on models trained with real data only, synthetic data only, or a combination of the two – what is preferred?

Overall, I find the method presented to be groundbreaking and believe it will significantly contribute to the field of bacterial cell segmentation. The manuscript shows great potential, but requires further changes to the language before I can recommend publication in PLOS Computational Biology.
