# Supplementary material for: Segmentation of dense and multi-species bacterial colonies using models trained on synthetic microscopy images
Source: PLoS Comput Biol. 2025 Apr 4;21(4):e1012874. doi: 10.1371/journal.pcbi.1012874 (PMC11970677; doi:10.1371/journal.pcbi.1012874)
Supplement: Attachment — Submitted filename: PLOS Comp Bio Response to reviewers.pdf [file pcbi.1012874.s003.pdf]

Response to reviewers:

Journal: PLOS Computational Biology
Original Manuscript ID: PCOMPBIOL-D-24-01793
Title: "Segmentation of dense and multi-species bacterial colonies using models trained on synthetic microscopy images"
Author(s): Hickl, Vincent; Khan, Abid; Rossi, René M.; Silva, Bruno F. B.; Maniura-Weber, Katharina.

We thank the reviewers for their thorough and constructive feedback. We have addressed all reviewer comments and modified the manuscript accordingly. The scope of the paper has been clarified through a major revision of the Discussion section, which better describes the method's strengths, limitations, and how we believe it will prove useful to the community. Additionally, we have extended our assessment of our segmentation models and added further quantitative comparisons to models from the literature. We have also updated some figures for content and clarity, and fixed any minor issues found in the text. Our detailed response to each reviewer comment can be found below.

Reviewer 1:

• 23-24: Great summary of the problem

We thank the reviewer for the positive feedback.

• 45-46: segmentation alone would never be expected to distinguish between species, only a separate classification network would do this. But yes, we absolutely want to train models that can segment any set of species simultaneously regardless of cell-to-cell variation in signal or morphology.

We agree that a single segmentation model cannot perform multi-species classification. In this case, however, since multiple models are trained to only detect bacteria with particular morphologies, combining these models results in a program in which multiple segmentation masks can be combined to simultaneously identify and distinguish between cells of multiple species.

We have amended the sentence as follows (lines 45-48):

"The mechanical interactions between multiple bacterial species at the microscale remain poorly understood, partially because there are few available analysis methods that can perform single-cell segmentation and distinguish between strains based on morphology alone."

• 64: very exciting promise!

We happily agree.

• 71: what does this mean? Back-diluted and grown to 0.2?

The culture, initially grown to the late exponential phase (OD > 1), was then diluted in fresh media to OD = 0.2, before being further diluted 50 times.

To clarify, we rewrote this and the next sentence as follows (line 74-76):

"The culture was supplemented with fresh medium to dilute it to OD = 0.2, and then further diluted 50× in medium, resulting in identical starting concentrations across experiments."

• 145: a great point, and it is good that it is made both for GAN and U-net

Thank you.

• 167-175: this is an unexpected approach. I would have thought that training to exclude some cells and include others would cause the model to not learn very well. It makes sense that a number of cells would be false positives, because Omnipose does some stretching of the images and crop reflections to simulate varied morphologies. I think that a separate model trained just for one-hot encoding of species used in conjunction with a global segmentation model would yield fewer false positives.

The ability to train segmentation models to only detect a particular subset of the objects in an image could be very useful for the study of bacteria in more diverse and clinically relevant settings. We originally tried our current approach to see if this would be possible using only the type of segmentation model already provided by Omnipose. While the approach suggested by the reviewers is promising, it would add a further complication to our workflow which is beyond the scope of this paper. The fact that simply training segmentation models to only detect rod-shaped or circular objects is as effective as shown here (true positive rate of ~90%), makes the approach extremely useful for our own work, and therefore worth sharing with the community. In particular, we hope that the effectiveness of this relatively straightforward approach could make tools like Omnipose more approachable to life scientists studying diverse systems who may have less experience using machine learning. Nevertheless, we agree with the reviewer that the use of an additional model trained for one-hot encoding would be worth attempting in the future.

We have added the following to our discussion (lines 469-473):

"In particular, an interesting alternative is to train a single segmentation model on synthetic images to segment all the cells in the images, and then separately train a classification model (for example using one-hot encoding) to identify the species of each cell. [...] Since the method presented here is rapid, versatile, and only requires the training of neural networks using widely available and well-established methods (cycleGAN and Omnipose or other similar segmentation algorithms), the barrier to entry is minimal, making it an ideal choice even for researchers with minimal computational experience."

• 180: PQ is very similar to the Jaccard index. This should probably be mentioned. It still has a maximum of 1, but the false negatives and false positives have half the effect as IOU cutoffs increase. Would love to see a PQ vs IoU cutoff curve.

To our understanding, the term Jaccard index is often used synonymously with intersection over union (IoU), both as a similarity coefficient between objects in images (which is the way IoU is used in this paper), or as a similarity coefficient between sets of discrete objects, in which case it equals TP/(TP + FP + FN). This double meaning could make the term slightly confusing in the context of our study. The latter case is indeed very similar to the recognition quality (RQ), which is one of two factors that make up PQ. The RQ is actually the same as the F1 score, a common alternative to the Jaccard index.

We added the following text to the methods section (line 194-196):

"The recognition quality (RQ) is also known as the F1 score. It is similar to the Jaccard index, another similarity coefficient in which false positives and negatives have a stronger effect."

As suggested, we have added a PQ vs IoU cutoff curve in Figure S5 of the supporting information, along with a paragraph describing how the quality of both segmentation models (the synthetic model and Bact_fuor_omni) varies with IoU cutoff:

"To quantify the overall quality of the segmentation models as a function of IoU cutoff, the panoptic quality (PQ, defined in the main text), is calculated for different IoU cutoffs, as shown in Fig. S5E. For the synthetic model, PQ drops off sharply above an IoU cutoff of about 0.7. For Bact_fluor_omni, this drop-off begins around a cutoff of about 0.55. For all values of the IoU cutoff, PQ is significantly larger for the synthetic model. This result shows that, if highly accurate segmentation masks are required, both models should be used with caution, but the synthetic one provides better accuracy for the noisy images acquired in this work."

• 192: a very clear and reasonable set of parameters for the image analysis,

Thank you.

• 261: I wonder how important modeling cell orientation at a global scale is. No doubt, we should have all kinds of interfaces represented in the dataset, but I would love to see models trained with and without global structure to see if there is anything gained by making this constraint in the image. My hunch is that it is more important to model ambiguous cell interfaces that occur from division.

One of the central goals of our research is to quantify self-organization, particularly orientational ordering, in bacterial colonies. It is thus paramount that we can rely on any segmentation we use to measure cell orientations in an unbiased and robust manner, so that our analysis accurately captured how bacteria align (or fail to align) under different conditions. Thus, in our case, creating a training dataset with varying degrees of orientational order is a precaution to ensure our segmentation algorithm is not biased to produce masks that exhibit a particular degree of alignment. In general, we agree with the reviewer that this precaution may be unnecessary for other applications. We have edited the methods section to make this clearer (line 112-115):

"Images with varying degrees of alignment were produced in each dataset to improve realism and ensure the training data for the segmentation model were not biased towards highly aligned or randomly aligned cells, which is important for the analysis of bacterial self-organization below."

Future work could be done to determine the precise effect of many different properties of the training data, including cell alignment, on the resulting segmentation model. However, such an in-depth analysis lies beyond the scope of this particular paper.

• 262-270: how do we know that the simulated cells match the cell boundaries? I think that a test against the symbac fluorescence model is critical (https://doi.org/10.1038/s44303-024-00024-4), as that was specifically designed to be physiologically accurate.

We agree that a comparison against a model trained on images from SyMBac would be appropriate to include in this paper. We have obtained the 'retrained' model from the authors of this work and have included it in the analysis for Fig. 3 and a new section of the Supporting Information (SI). While the SyMBac model generally outperforms Bact_fluor_omni (trained on real images), its tendency to oversegment cells in our images causes it to fall short of our model trained on images processed by cycleGAN. Thus, the main conclusions of the paper remain unchanged.

To better quantify how well the segmentation models matches the cell boundaries, we have conducted a detailed analysis of how accurately cell positions, orientations, aspect ratios, and cell areas can be measured using the model trained on synthetic images. We compare the masks from this model as well as bact_fluor_omni with hand-drawn masks on a representative set of test images. This analysis is described in detail in the newly added section of the Supporting information (Figure S5 in particular). Please also see our response to the next comment directly below for a summary of our findings.

• 307: IoU of 0.5 is frankly a meaningless cutoff. Only 0.8 or higher should be used for true positives. Fig 3E shows that most of the IoUs are >0.5, so this should not affect the qualitative results of the paper, but it is an important quantitative distinction.

We cannot fully agree that a cutoff of 0.5 is meaningless. In general, the accuracy required from a segmentation algorithm depends on the characteristics of the cells to be analyzed. Therefore, the IoU cutoff should also be chosen accordingly. For our research, we are primarily interested in extracting cell positions, orientations, and aspect ratios from our images. Additionally, cell areas should be measured accurately enough to measure cell packing fractions.

Initially, we chose the cutoff of 0.5 for two reasons: 1) it guarantees uniqueness of true positives, meaning that a given ground truth cell cannot have two candidate cells above the cutoff. 2) We believe the candidates above this threshold gave sufficiently accurate measurements of the above characteristics.

To test the validity of this choice, we have now performed a detailed analysis of how accurately cell positions, orientations, aspect ratios, and areas are measured by the segmentation algorithm relative to the ground truth. A section has been added to the Supporting Information (SI) describing this analysis. In short, above an IoU of 0.5, cell positions are measured to within a typical cell width (0.75 μm) and cell orientations are measured to within <π/10 radians. Both cell aspect ratios and areas are accurate to within <20%. For cell aspect ratios and areas, the masks from the synthetic model are more accurate than those produced by bact_fluor_omni even when controlling for IoU. The models perform about equally well when controlling for IoU in terms of cell position and orientation. For the purposes of quantifying bacterial self-organization, we believe this accuracy is sufficient.

As described above, we have also included a PQ vs IoU cutoff curve to demonstrate the effect of changing the cutoff on the estimated quality of the segmentation. The model trained on synthetic images outperforms bact_fluor_omni and the symbac model for all IoU cutoffs.

We also emphasize that in some images (including the test images used for Fig. 3), unambiguously annotating the images by hand is not trivial. Due to the lower signal-to-noise and the dense packing of cells, some of the hand-drawn labels are effectively best guesses as to where the cells really are. Therefore, it is not guaranteed that differences between the masks produced by a segmentation model and the hand-drawn ones are bona fide errors, which likely results in us underestimating the accuracy of the model. For example, our new analysis shows that candidate cells at high IoUs tend to be smaller (eroded) than the hand-drawn labels. Since we expect a large PSF when imaging through a layer of PDMS, it could be that the hand-drawn labels are actually overestimating the true size of the cells, and that the masks produced by the model are more accurate than we calculate here. Whether this is a desirable feature of a segmentation model is perhaps unclear, but it highlights the difficulty associated with evaluating the segmentation quality, as well as the limitations of hand-annotated training data for datasets such as ours.

In addition to the new section in the SI, the following has been added to the main text (lines 315-320):

"This IoU threshold guarantees that at most one true positive exists for each ground truth cell. The accuracy with which cell positions, orientations, aspect ratios, and areas can be measured was analyzed as a function of IoU (see Supporting Information for detailed analysis). It was found that these important characteristics can be reliably measured using a threshold of IoU > 0.5, as shown in Fig. S5."

- 309: the most recent state of the art model is actually the aforementioned symbac model (granted, this was only published in September). This should be shown in addition to or instead of bact_fluor_omni, which was shown to dilate cell masks (this was an artifact of elastic local registration in the training set).

As stated above, we added a detailed comparison to the SyMBac model to the manuscript. The results of this analysis are included in Fig. 3, and described in lines 340-365:

"For the test images obtained here, the synthetic model trained on images processed by cycleGAN outperforms the model trained on images from SyMBac. These two models produce equal proportions of true positives and false negatives. However, the SyMBac model produces a large number of false positives, suggesting significant oversegmentation of cells. As a result, the PQ is significantly better for the model trained on images processed by cycleGAN. The mean IoU of candidate cells from the `synthetic model' is 3% higher than that of the SyMBac model, on average. When controlling for IoU, measurements of cell positions, orientations, and aspect ratios are comparable across these two models trained on different synthetic images, but measurements of cell areas are more accurate using the model trained on images processed by cycleGAN, as shown in Fig. S5. It should be noted that the segmentation model trained on images from SyMBac is intended to be a bespoke model for a particular set of experimental and imaging conditions, which may differ from the ones described here. The PQs of all three models decrease when the IoU cutoff is increased, as shown in Fig. S5E. For all IoU cutoffs, the model trained on processed synthetic images performs better than the two models from the literature."

• 462: I think that the tactic of cell simulation will indeed be key to developing cell classification based on signal and morphology. However, training separate segmentation models is likely not the best option. Training one-hot encoding models will be more efficient and result in fewer false positives, and this technique of simulation is absolutely critical to training models to do so (as the number of classes with change with the number of prediction species). The possibility that alternative models would be better for this task is discussed in 470-471.

As mentioned above, we have elaborated on this possibility in the discussion (lines 488-492):

"In particular, an interesting alternative is to train a single segmentation model on synthetic images to segment all the cells in the images, and then separately train a classification model (for example using one-hot encoding) to identify the species of each cell."

Fig 1:

• There are a LOT of artifacts in the simulated images. Linear artifact probably a result of tiling and lack of blending. But there are other artifacts in the PSF.

We agree that there are artifacts in some of the images processed by cycleGAN. In particular, the processing step seems to occasionally result in (faint) cells being added to the synthetic images, which could be problematic since these are not present in the corresponding masks. However, we currently believe this issue is not particularly problematic, as the number of cells missed (false negatives) by the segmentation model trained on these images is low. We believe that a potential solution is to take greater care to ensure the cell concentrations in the synthetic and real images used to train cycleGANs are the same, or to train separate GANs for very densely packed images. The following text has been added to clarify this potential limitation and describe how we believe it could be overcome (lines 455-460):

"The use of GANs causes processed synthetic images to occasionally contain artifacts such as additional, faint cells that are absent in the original masks, as seen in Fig. 1C. Ensuring

that the cell concentration in the raw synthetic images is high already minimizes this problem, as demonstrated by the low false negative rate, but further improvements to cycleGAN training may be necessary to remove the hallucinated cells altogether."

• I suggest using the ncolor package to use shades of green for Pa and shades of red for Sa.

We have chosen to avoid red and green together to avoid confusion with the colors used in Fig.3C (and because it is not ideal for colorblind readers). We believe bright green and cyan offer good contrast in images with both light and dark backgrounds, allowing us to use consistent coloring throughout the paper.

FIg 2:
• The IoU panel only shows one type of offset that would cause less than 1. I suggest showing other kinds of errors, as I doubt the most relevant error here for bact_fluor_omni is the offset. I think it would be a dilation.

The diagram has been edited to simultaneously show three kinds of errors: translation, dilation, and rotation.

FIg 3:
• I would maybe use one shade for masks that stayed the same and red etc. for masks that changes substantially, so that it is clear what was correct. Or maybe a difference mask.

We have changed the cells colors in Fig. 3C as suggested.

Data: there is an oversight in the data availability. The masks and binarized images are in the synthetic images folder on zenodo, but not the actual cycleGAN output.

We have added processed synthetic images to the Zenodo repositor and updated the link accordingly.

<u>Reviewer 2:</u>

In this manuscript, the authors present an exciting and novel method for training a cell segmentation model: simulating cell geometries to create artificial masks, applying an image-to-image translation using a cycleGAN to generate synthetic images similar to real images, and then training image segmentation models which have only used synthetic images and masks as ground truth data. Previous methods to generate synthetic images have involved complex simulations of cell growth and imaging conditions; the simulations for the current method are simply based on cell morphology, and the method is amongst the first of its kind for bacterial cells.
This method is highly significant because i) the use of synthetic images and annotation as ground truth data solves the issue of time-consuming human annotation as a major bottleneck of model training, ii) there is no bias in annotation, and iii) synthetic images can be adjusted to accommodate various imaging and sample conditions, amongst other advantages.
In its current state, I feel that the manuscript presents more of a proof of concept of the method, which will be highly adaptable for various experiments. An experimentalist would be interested in

using the trained models, which are not made available. Thus, I believe it is important for the language of the manuscript to be more precise.

We thank the reviewer for their thorough reading of the manuscript and thoughtful comments. We have addressed all their comments below and improved the paper accordingly.

Comments:

1. It would be helpful to make a stronger language distinction between “masks”, “raw synthetic images”, “synthetic images” and “processed synthetic images” within the manuscript. For example, l. 52 uses “synthetic microscopy images” when I believe this should be referring to masks. L. 167 refers to synthetic images, but I believe these would be the processed synthetic images. I think l. 275 is referring to processed synthetic images. This language change would make it easier for readers to understand and recreate the process.

Throughout the paper, we have ensured that synthetic images are described as "raw" or "processed" when appropriate for added clarity. The instance on line 52 (now 55) is correct. While masks are produced alongside the images, they are not used as inputs for cycleGAN training. There and elsewhere "synthetic images" is used in a general sense, without referring to raw or synthetic ones in particular.

2. Throughout the paper, an IoU>0.5 is used. However, this is very low, as IoU>0.8 is highly preferred for accurate predictions (see Avoiding a replication crisis in deep-learning-based bioimage analysis - Laine 2021).

For this paper, the purpose of the IoU is to assess whether a given candidate cell produced by a segmentation model matches the ground truth with sufficient accuracy. What is considered "sufficient" naturally depends on the intended application, that is, the analysis to be carried out using the segmentation masks. For our research, and that of others studying bacterial colonization of surfaces and self-organization, the most important parameters are cell positions, orientations, sizes (areas), and morphologies (in our case aspect ratio).

We have added a detailed analysis of how well segmentation masks allow us to measure these quantities as a function of IoU to the supporting information. As described in more detail above in response to another comment, it shows that for IoU>0.5, all of these properties can be measured with the desired accuracy. We do not in the SI that for other applications where more precise measurements of cell outlines are required, a higher IoU cutoff may be appropriate, and the models trained here should be used with caution. That being said, the model trained on our synthetic images measures these quantities as accurately, and often better, than the pretrained model for Omnipose.

Note that the article mentioned by the reviewer only briefly mentions 0.8 as a criterion for good agreement, and even cites 0.5 elsewhere as a common cutoff for determining which objects count as "detected," seemingly without criticism (Box 1). This article is an excellent resource, and we have added it to our citations (number 17, line 27).

We have also added a PQ vs IoU cutoff plot to evaluate the quality of the segmentation model for different cutoffs (Figure S5E).

3. Results: The meaning of the IoU metric in the context of this paper is ambiguous. As IoU is found from comparing results on a pixel level, images of cells with noisy or poorly defined boundaries could result in very different IoU values. Furthermore, since the Omnipose fluorescence model was trained with cytoplasmic and membrane fluorescence while the synthetic model is trained and tested on nucleic acid stains, should the performances be able to be compared?

The first statement by the reviewer here is an important point, which we agree needed mentioning and underscores the value of using synthetic training data. The IoU values here are calculated relative to 'ground truth' masks drawn by hand, corresponding to our best attempt at manual segmentation and intended to provide the necessary information to analyze the monolayer. The purpose of training a segmentation model, then, is to automate this process and remove human bias, without sacrificing accuracy in the downstream analysis. The use of synthetic training data is ideally suited to this task, as it allows us to create a program which divides the images into individual cells using prior knowledge of their shapes and morphologies. Such a program cannot be created using hand-annotated training data, which necessarily requires cells to be distinguishable by eye.

Nevertheless, the hand-annotated images had to be used to evaluate the quality of the segmentation simply for lack of any alternatives. Given that, to our knowledge, existing segmentation models from the literature do not provide accurate segmentation of these images (as exemplified by Bact_fluor_omni), any new segmentation model can only be compared to a manual 'ground truth.' This is a fundamental restriction when developing new segmentation techniques, and not unique to our study. As a result, we believe that IoU, combined with counts of true positives, false negatives, and false positives, are the best way to quantify the quality of our segmentation models.
To clarify the above, and the fact that differences between manually drawn labels and those from trained segmentation models are not necessarily 'errors' in the usual sense, the following has been added to the SI:

"It should be noted that, because imaging through the sheet of PDMS produced images with a low signal-to-noise ratio, hand-drawn masks are not guaranteed to correspond perfectly to the ‘real’ cells. For very densely packed regions especially, individual cell boundaries can be hard to identify, meaning differences between the hand-drawn labels and the labels from the segmentation models may not necessarily be errors, but rather the result of a different attempt to divide the bright regions in the image into discrete cells."

At the time of writing this manuscript, the pretrained model for Omnipose was the most suitable model for our images available to us from the literature despite the differences in how the training data were prepared. Given the general strength of Omnipose, we expected it to segment our images reasonably well. In light of the limitations described above, we do

not believe a model trained on images with different staining techniques would result in a significantly better segmentation model, as long as the training data do not share the noise levels and (at times) uncertain cell boundaries present in our images. As described in response to reviewer 1, we have also added a detailed comparison of our model's performance to a segmentation model trained on synthetic images from SyMBac. Again, our model outperforms the SyMBac model, since it is made specifically for the images obtained from our experiments.

4. The section headers in the Results section could be stronger. For example, “Segmenting dense P. aeruginosa monolayers” could mention outperforming existing models.

We revised the last four headers as follows:

"Rapid training of segmentation models using synthetic images and image-to-image translation"

"Accurate segmentation of dense *P. aeruginosa* monolayers using synthetic images"

"Quantifying bacterial self-organization in dense *P. aeruginosa* monolayers"

"Simultaneous segmentation and classification of multi-species colonies"

5. The resulting masks of the multi-species segmentation are very poor (Figure 5)—for example, some of the spherical or rod-shaped cell masks have sharp points. Is this a result of poor image quality, lacking training data for the model, or another factor; how could these results be improved?

We believe these features of the masks are primarily the result of the somewhat larger pixel size in the brightfield images (~0.2 μm) and the small size of the cells (<0.5 μm radius). The shape of the masks appears smoother in higher resolution images, as shown in the cases where confocal microscopy was used. For cells of this size at this resolution, the cells in the images sometimes simply appear to have sharper features, so we do not think it is very problematic that the segmentation model occasionally generates masks accordingly.

6. The multi-species capability seems to depend on the model being trained to recognize/ignore different cell morphologies. Would the authors expect this method to be able to differentiate between different species with similar morphologies (ex, P.a. and E. coli in the same image, both rod-shaped)?

If two species have precisely the same morphology (e.g. two mutant strains of a species), we would not expect this approach to allow us to distinguish between them. However, it remains to be seen how far the bounds of our method can be pushed to distinguish between cells with similar morphologies, such as the given example of *P. aeruginosa* and *E. coli*. While both species are rod-shaped, *E. coli* are larger on average, which could be sufficient to distinguish between the two with some accuracy. Naturally these finer differences would

need to be reflected in the synthetic images as well. We hope to be able to test these and many similar ideas in our future work.

The following addition to the discussion addresses this point (line 463-467):

"However, the approach presented here cannot distinguish different bacteria strains with identical morphologies (e.g. mutant strains of a single species), and further testing will need to be carried out to determine how accurately bacteria of similar morphologies (e.g. two rod-shaped species of different sizes) can be classified."

MINOR COMMENTS:

1. The method is mentioned to work for both confocal and brightfield microscopy, however, the manuscript focuses heavily on the results for confocal microscopy. The authors support the idea of only using a single fluorescence channel/stain and using cell morphology to distinguish cells. As brightfield images are subject to less noise and photobleaching, in addition to having better defined boundaries, I would have thought that demonstrating the method for brightfield imaging would make a stronger case. Furthermore, the method was not shown for dense brightfield images, where cell contact would present a larger issue as compared to SYTO 9 stains, which are less likely to have overlap. I would also like to see an example of the processed synthetic brightfield images.

We have replaced the brightfield image used in Fig. 5 to one where cells are more densely packed, showing our approach is effective on such images as well.

In general, we believe demonstrating that our method works for the imaging method with more noise and photobleaching is to make a stronger case for its merits overall. In this case, we agree that the demonstration is more challenging for confocal microscopy, largely due to the additional sample preparation requirements and (at times) uneven staining between different cells. Thus, we focus on demonstrating that our approach is effective even in this case.

We have added an additional figure to the supporting information showing several representative synthetic brightfield images of mixed colonies before and after processing by cycleGAN (Figure S4).

2. Verb tense in Author summary: We apply… and found…

We made all tenses in the author summary consistent.

3. Author summary: "multiple imaging methods, including both brightfield and confocal microscopy" - were other imaging methods investigated?

No other methods were investigated. The sentence has been revised to:

"We apply this technique to images containing multiple species of bacteria and find that it is very effective for both brightfield and confocal microscopy."

4. May add this reference regarding synthetic images to l. 33 https://www.nature.com/articles/s44303-024-00024-4

Done (line 35).

5. L. 134: wass -> was

Done (line 139).

6. L. 147: compared it to -> compared to

This sentence was rewritten (see next comment).

7. L. 147: Suggest rewriting the sentence for clarity, such as: The FID between the real dataset and the processed synthetic dataset was used to quantitatively compare sets of synthetic images to real images.

To improve clarity, we rewrote this sentence and the previous one as follows (line 150-153):

"To obtain the best model in our training process, a dataset of processed synthetic images was constructed every 10 epochs. Then, the Fr\'echet inception distance (FID) between the real dataset and each processed synthetic dataset is calculated."

8. L. 152: generated by the cycleGAN... using the Omnipose package. (add the, remove for Python)

We added "the" as suggested (line 157). However, we feel it is useful for the reader to know which programming language was used for each part of the methods.

9. L. 173: “false negative positive rate” is slightly confusing.

Removed "negative" (line 177).

10. The language in l. 187-191 could be more precise.

We improved our explanation of the terms that make up the panoptic quality as follows (line 191-197):

"The first term in the PQ is sometimes called ``segmentation quality" and measures how well true positives match with the corresponding real cell at the pixel level, on average. The second term is the ``recognition quality," which measures the model's ability to correctly find

true positives. The recognition quality (RQ) is also known as the F1 score. It is similar to the Jaccard index, another similarity coefficient in which false positives and negatives have a stronger effect. Thus, PQ provides a comprehensive metric for evaluating the performance of a segmentation model."

11. L. 214: Could be more clear with the term "associated". Is the synthetic image directly generated from the mask/the mask generated from the synthetic image?

We changed the sentence as follows (lines 220-221):

"A `mask' is produced by the model alongside each raw synthetic image to encode."

12. L. 250: Don't see clusters of cells in Fig2 bottom right.

As mentioned above, we have replaced the brightfield image in Fig. 5 to show more clustered cells. We have changed the figure reference in the text accordingly (lines 256-257).

13. Fig 3 caption: "intersection over union (a common measure of how accurately cells are identified during segmentation)" This language could be more precise. IoU measures accuracy on a pixel-level rather than just measuring if cells are or are not identified.

We updated the explanation of IoU in the caption as follows:

"...which measures how accurately cells are identified by comparing candidate cells in the segmentation mask to the ground truth at the pixel level."

14. Fig 4: Color is very important in this image, however, some cells have pixels of multiple colors.

We agree that color is crucial here, but upon close examination, we cannot find any cells with multiple colors. Could this be caused by some kind of compression or file conversion in the submission portal?
The code we use to create images such as Fig. 4 A assigns a single color to each cell and fill the corresponding mask in accordingly. We double checked this code an created the image again but notice no difference to the previously submitted version.

15. Fig 4 caption: analyzed here include images -> analyzed here are extracted from images

Change was made as suggested.

16. L. 294: could mention undersegmentation

Over- and under-segmentation are both mentioned in the following paragraph (line 311-312), since they our analysis suggests they are about equally common.

17. L. 313: models like it -> similar models

Done (line 326).

18. L. 327: provided -> provides

Done (line 359).

19. L. 351: decrease -> decreases

Done (line 388).

20. Section "Single-cell statistics from dense Pseudomonas monolayers": This section could benefit from a structure more like "we expect… we find/show…" It was ambiguous whether the first line of each paragraph was a conclusion from the data or an expected relationship.

Each paragraph is structured such that it starts with a topic sentence stating the main result, followed by a more detailed description of the result and how it was obtained. To clarify this format, we added the phrase "it was found that" to the first paragraph of the section (line 366).

21. L. 419: possibly missing the word 'high' before "cell densities"?

Indeed, the word "high" has been added (line 526).

22. L. 436-444: I found this section confusing. Wasn't the model trained to identify circular cells for S.a., while the model to identify non-circular (rod-shaped?) cells for P.a.? The P.a. model is also referred to as the P. aeruginosa model. It may be helpful to have consistent names for these models.

The reviewer is correct about which bacteria each model was made to detect. The model trained to identify circular cells (S.a.) may occasionally fail to detect a real S.a. cell if it deviates too much from circularity. This cell may then be detected by the model trained for rod-shaped cells (P.a.), hence the increased false positive rate for the P.a. model as described. To make this point clearer, we have edited the section as follows (lines 444-448):

"While S. a. cells were modeled as perfect circles, they often appeared more oval-shaped or amorphous in real images (particularly during cell division), leading to a higher rate of false negatives from the model trained to identify circular cells and false positives from the model trained to identify non-circular cells (since the latter model would erroneously detect S. a. cells with higher aspect ratios)."

23. Citations: bacterial names should be italicized for ref.s 6, 9, 25, 26, 27, 29, 34

Done.

24. Supplemental Material: c ycleGAN -> cycleGAN

Done.

25. Perhaps beyond the scope of this manuscript, but it may be interesting to make a comment on models trained with real data only, synthetic data only, or a combination of the two – what is preferred?

We agree both that this would be interesting, and that it is beyond the scope of this article. Our original goal was to show that synthetic training data processed by cycleGAN could be a good substitute for hand-annotated (experimental) training data. In the process, we discovered that models trained on this synthetic data could actually perform better than those trained on the real models. We believe this surprising result would, by itself, be of great interest to others working on image segmentation problems, particularly involving bacteria.

If, in other systems, synthetic images produced this way prove insufficient to yield the desired level of segmentation accuracy, supplementing them with real training data would be very interesting. To highlight this point, we added the following to the discussion (line 470-477):

"Slight differences between synthetic images and real images will always remain, potentially limiting the accuracy of segmentation models trained on synthetic data alone. Therefore, it would be interesting to train models on a combination of hand-annotated real images and synthetic images to determine whether doing so can further improve their accuracy. Transfer learning could be an especially interesting approach, where a model is pre-trained on large synthetic datasets first and refined using real data. The pre-trained model could be used to greatly speed up annotation by producing masks which only need to be corrected slightly by hand."

Overall, I find the method presented to be groundbreaking and believe it will significantly contribute to the field of bacterial cell segmentation. The manuscript shows great potential, but requires further changes to the language before I can recommend publication in PLOS Computational Biology.

Reviewer 3:

This article proposes a strategy for training microscopy image segmentation models from transformed synthetic binary masks. This manuscript could have been potentially interesting but I have several major comments:

We thank the reviewer for their feedback. Based on their comments, we believe it is necessary to clarify precisely what the main results of the paper are (and what they are not), and how it fits into the existing literature. We have significantly rewritten the Discussion section to better describe the strengths and limitations of our method, and how future work can build on our findings. We describe specifically how each of the reviewer's comments was addressed below.

1 - In my opinion, this work does not demonstrate that synthetic image production is strictly necessary to improve CNN-type model training. Indeed, there are no results showing that a simple modification of synthetic masks (such as Gaussian filter and noise) is not sufficient to train a CNN model and thus provide better results than pre-trained models.

We believe that our results, particularly those in figure 3 and the newly added figure S5, show that synthetic images, as produced here, can produce segmentation models that outperform existing ones available in the recent literature. We do not claim that the use of synthetic images is strictly necessary to train better segmentation models. However, synthetic images offer the significant advantage of eliminating the need for hand-labeled training data, saving many hours of tedious work. Thus, even if our model only performed equally well to models trained on hand-annotated data, our approach would be highly advantageous and therefore worth sharing with the community.

Naturally, we are not the first to use synthetic images to this end. Other researchers using images produced by various models or simulations have shown that these synthetic images must then be modified significantly to match the optical properties of the experimental setup (see Hardo et al., 2022, Zhang et al., 2022, or Toma et al., 2022 for examples). These modifications require either modeling the image acquisition apparatus to compute a point spread function (PSF) or measuring the PSF experimentally. As a result, we are confident that for our images, simply adding noise and/or a Gaussian filter would not be sufficient. We did once attempt to train a segmentation model on the unprocessed ('raw') synthetic images with only the addition of some Gaussian noise, and the result was too inaccurate to warrant publication.

With these considerations in mind, it becomes clear that the use of cycleGAN provides a unique advantage by processing synthetic images to resemble experimental ones. This approach avoids the complex modelling or additional experiments required to obtain a PSF, making it easy to train models for multiple imaging modalities and experimental conditions

with little additional effort. Moreover, we believe this approach is more accessible for microbiologists with a less computational background who may find the prospect of extensive modelling daunting.

The new subsection of the discussion " Key advantages of this method" (lines 418-440) specifically describes these strengths of our method.

2 - This work does not demonstrate that training with the GAN-transformed synthetic masks (circles and rods) improves results obtained for different morphologies (crescents, chains, curved rods, etc.). Moreover, if the user must generate their own synthetic binary masks for the morphologies of interest, no solution is proposed in this direction. These two essential aspects for applying this method to real cases of morphological diversity are not discussed. And precisely, the authors insist on the need for universal solutions for bacterial mixtures in real community study conditions. The work presented in this article absolutely requires results obtained with real bacterial images presenting a true diversity of shapes (Caulobacter, Anabaena, Streptococcus, Spirochaetota, morphological mutants etc.).

We agree with the reviewer that including additional cell morphologies will be critical in the future. However, we believe this is beyond the scope of this paper. In particular, we do not agree that a universal solution able to perform segmentation in all possible systems is necessary, nor did we make such a claim in the paper. In fact, this method's use of cycleGAN enables precisely the opposite: since the processing and training with synthetic images can be done relatively quickly (a few days of highly automated computation), bespoke segmentation models can be created at little marginal cost for different experimental setups.

To model bacteria with different morphologies, some tools are already readily available in the literature. For example, Jelli et al. (DOI: 10.1111/mmi.15064) Have published a method for drawing toroidal cells such as *V. cholerae* using an approach somewhat similar to ours. Using these and other simulation tools (e.g. those found in https://doi.org/10.7554/eLife.72187, or https://doi.org/10.1038/s41467-019-10311-z), other researchers can produce 'raw' synthetic images without needing to create entirely novel software. Ultimately, the aim of this paper's last section is to demonstrate that the approach of training separate segmentation models for different cell morphologies in a single image can be an effective way to achieve both single-cell segmentation and classification of morphologically distinct cells. Future work can then focus on pushing the limits of this approach to determine which cells morphologies can be distinguished with high accuracy.

The new subsection of the Discussion "Current limitations and potential extensions" (lines 439-502) specifically describes how our method is complementary to other recent advances. We also clarify how this approach is particularly well-suited to creating specialized segmentation models for different applications (line 455-460):

"The use of image-to-image translation via cycleGANs greatly simplifies the creation of realistic synthetic images suitable for the training of segmentation models. The only knowledge required for this step is the approximate geometry and concentration of cells in the images;

no explicit modeling of the biology, physics, or optics of the experiment is required. These features make creating highly specialized segmentation models for different experimental setups highly efficient. In general, this adaptable method could be used by researchers from a wide variety of backgrounds for image analysis applications in the life sciences and beyond."

3 - The authors base their comparison on the improvement in results compared with a single example of a pre-trained model, namely "Bact_fluor_omni". This comparison is not sufficient for two reasons: firstly, only a pre-trained model for segmentation from fluorescence images was tested, but many light microscopy explorations use phase contrast images as the source for cell segmentation (the omnipose package provides a pre-trained model for phase contrast). The GAN process of synthetic image should be able to mimic the microscopy phase contrast image in order to train a model with. Secondly, the only type of pre-trained model tested is a CNN-type model, whereas currently models based on "transformers" seem to have a better ability to adapt to the diversity of objects in the image (this is illustrated in these articles which are not mentioned in this work: Ma et. al Nat Methods. 2024 June ; 21(6): 1103–1113. doi:10.1038/s41592-024-02233-6, Gihun et al. Mediar: Harmony of data-centric and model-centric for multi-modality microscopy. In Proceedings of The Cell Segmentation Challenge in Multi-modality High-Resolution Microscopy Images, volume 212, pages 1–16, 2023).

We are aware of the papers mentioned by the reviewer, which are groundbreaking advances in image segmentation. However, they represent a fundamentally different approach to ours, making a direct comparison difficult. The methods in all three articles describe attempts at achieving cell segmentation in a wide variety if multi-modal image using weakly supervised segmentation, meaning the training data include more unlabeled than labeled data. In other words, it is an attempt towards a 'universal' segmentation solution, as mentioned by the reviewer in another comment above. By contrast, our method is designed to allow researchers to quickly and efficiently create highly specific, bespoke segmentation models to match their particular experimental conditions.

These two approaches are highly complementary, as specific models are invariably needed for special cases where universal models fail. In particular, since universal models are trained to detect cells of many different shapes and sizes, they are ill-suited to the challenge found in our data, where cell boundaries are often difficult to identify, and distinct cells may be touching or overlapping. For such cases, the use of synthetic images allows us to use prior knowledge of our system to train a model to detect cells of particular morphologies. Moreover, any method intended to segment many different cell types is ill-suited to the task of multi-species classification unless a separate classification model is also trained (we mention this possibility on lines 488-492). As a result, a direct comparison is only of limited value and lies beyond this paper's intention. It should be noted that the last paper cited also uses CNNs (from Cellpose specifically) rather than transformers.

The goal of comparing the performance of our model against Bact_fluor_omni is not to compare different types of models (such as CNN and transformers), but rather to compare

the use processed synthetic data to the use of real, hand-annotated images. Our approach is not intended primarily to be used for weakly supervised or unsupervised learning (although this would be an exciting extension!). Therefore, we do not think it is necessary to include a comparison between CNNs and other model types, including those in the works mentioned here.

By demonstrating the efficacy of our approach with both confocal microscopy and brightfield microscopy, we have already shown that the method can be used to create synthetic data for different microscopy techniques.
