# Supplementary material for: Segmentation of dense and multi-species bacterial colonies using models trained on synthetic microscopy images
Source: PLoS Comput Biol. 2025 Apr 4;21(4):e1012874. doi: 10.1371/journal.pcbi.1012874 (PMC11970677; doi:10.1371/journal.pcbi.1012874)
Supplement: Attachment — Submitted filename: PLOS Comp Bio Response to reviewers2.pdf [file pcbi.1012874.s004.pdf]

Empa
Materials Science and Technology

Response to reviewers:

Journal: PLOS Computational Biology
Original Manuscript ID: PCOMPBIOL-D-24-01793
Title: "Segmentation of dense and multi-species bacterial colonies using models trained on synthetic microscopy images"
Author(s): Hickl, Vincent; Khan, Abid; Rossi, René M.; Silva, Bruno F. B.; Maniura-Weber, Katharina.

Reviewer 1:

The responses to all reviewers was extremely thorough and made for substantial improvements to the manuscript.

> We thank the reviewer for this positive feedback.

A last weakness of this manuscript worth mentioning is that we do not have a head-to-head comparison of a segmentation model trained on cycleGAN images versus one trained on corresponding real images. The authors are careful to mention that the bact_phase_omni and SyMBac models are trained on distinct datasets not representative of the test dataset. We should of course expect that those models would perform worse than any model trained using real, representative data. The huge innovation in this work is that no manual annotation was required to train a new segmentation model on such a distinct dataset. However, the fact that it performs better than the other models is not particularly meaningful - a segmentation model trained on apples should not be expected to segment oranges. Instead of training cycleGAN on their own data, the authors could instead train on the same training datasets of the aforementioned models. This would eliminate the variable of train vs test data representation and show that it is possible to reach X% of the segmentation accuracy without manual annotation. [There is the subtlety that this experiment uses manual masks instead of generated masks ("synthetic images" in the manuscript), but generation of arbitrary cell morphologies is outside the scope of the paper.]The principal claim of this work is that the cycleGAN produces good enough training data for a segmentation model to be useful. How useful depends on the scientific question to be answered, and the authors demonstrate their use case well. Other use cases would require far higher segmentation accuracy, and this work does not give such a limit. Although the manuscript feels incomplete without this comparison, the advancement it demonstrates is sufficient for publication in my opinion.

> We agree with the reviewer that the extension to our paper they describe would be interesting, although it addresses a slightly different question than the one motivating our work. For researchers studying systems like ours, where imaging conditions are suboptimal and existing models from the literature (e.g. the Symbac model and Bact_fluor_omni) fail, the crucial choice they face is not whether to use a model trained on hand-annotated real images or synthetic images, but whether to attempt single-cell segmentation at all. For many researchers, the cost of producing a hand-annotated training set is prohibitively high, particularly if imaging conditions vary across experiments. As a result, important systems may not be studied quantitatively at the single-cell scale at all. Our paper was written with these cases in mind, to lower the barrier to entry and empower others with access to high-resolution imaging to perform more quantitative image analysis. Therefore, to train a cycleGAN

for synthetic image processing on a set of images that have already been hand-annotated and used to train a segmentation model is not as important to our study. In any case, we thank the reviewer for supporting the publication of the manuscript without this addition.

For that advancement to be useful to anyone, documentation must be written to guide users through training a cycleGAN on their own data. Hardware requirements, package versions, training image folder structure and file naming, etc. must all be specified in the GitHub README. A commented .py script or a jupyter notebook should be provided to run the training. Expected training times would also be helpful.

All the requested information, including the hardware used (with corresponding training times), required packages with versions, and folder structure have been added to the GitHub README. We also added a .yaml file – allowing users to easily duplicate the Python environment we used on their own machine. A training notebook was already provided ('cycleGAN bin_cross.ipynb'), but detailed instructions on its use and that of the other notebooks provided for FID score calculation and image generation have been added both to the README and to the documentation within each notebook.

Additionally, we have added example images to the GitHub repository for users to test their installation. As requested, the manually annotated test images used to compare models and to calculate the PQ vs. IoU curves have been added to the Zenodo repository.

As a last minor comment: “synthetic image” vs “processed synthetic image” is still confusing. Consider using “binary mask” or “generated masks” for the procedurally generated, binarized cell labels and reserve “synthetic image” for the output of the cycleGAN. This would be more consistent with existing literature.

We would like to avoid the use of 'masks' to refer to the synthetic images, as we believe it would lead to confusion with the label masks used to train the segmentation model. Similarly, we worry that 'generated' would be confusing here, since a 'generator' refers to the model trained as part of the cycleGAN to transform images from one domain to the other. While the 'raw' synthetic images are initially equivalent to binary masks, the cycleGAN code adds Gaussian noise, meaning the synthetic images ultimately used to train the cycleGAN were not simply binary masks.

We have added a paragraph to the methods subsection 'Synthetic images for segmentation model training' to better described the creating of the label masks for the synthetic images, and the precise meaning of 'raw' and 'processed' synthetic images (lines 122-131):

"Simultaneously, this program also produced a label mask for each synthetic image -- an integer array of the same dimensions as the image in which the set of pixels corresponding to each cell is assigned a unique integer value. These masks were later used to train the segmentation model. Initially, raw synthetic images were simply binary masks -- white cells on a black background. Gaussian noise (standard deviation = 0.1) was added to these images to ensure enough variability across the synthetic data set. The addition of noise was performed within the program used to the train cycleGANs, described below. Throughout

this paper, the terms `raw synthetic image' and `processed synthetic image' are used, respectively, to refer to these images before and after they are transformed by cycleGAN to resemble real images."

Reviewer 2:

Excellent work! This is very exciting and a helpful contribution to the field.

Thank you for the positive feedback.

Reviewer 3:

This manuscript is of real interest in the field of bacterial microscopy image segmentation. The authors have improved the manuscript taking into account the reviewers' remarks. The revised version highlights the strengths and limitations of this work.
But on the substance of the paper, it could have had a greater impact if the authors had diversified the morphologies of the synthetic images and if they had carried out phase contrast tests. Indeed, there is no evidence to suggest that the method described here improves results for morphologies other than rod morphologies. The authors consider that tests with fluorescence images (confocal and wide-field) validate their method for all microscopy modalities. I believe that tests with phase contrast images and the omnipose phase contrast model would have greatly increased the scope of this work and its interest for microbiologists who make extensive use of phase contrast.

We thank the reviewer for their feedback.

In our paper, we do not make the claim that our tests on fluorescence images validate the method for all modalities. We do, however, show that we can accurately segment cells in images of multi-species colonies from both confocal and brightfield microscopy. The task of separately segmenting cells with different morphologies in the same images (and thus effectively performing multi-species classification) is significantly more challenging than segmentation alone. Therefore, we believe that demonstrating that this segmentation and classification is possible using our method for multiple imaging modalities provides strong evidence of its versatility. Certainly, single-species segmentation would also be possible for brightfield microscopy, and, we are convinced, for phase contrast microscopy as well, since the two are similar. Additionally, our results on multi-species segmentation do show that our approach is effective for cells with different morphologies.

Therefore, we believe that performing additional experiments and training additional models to segment the same bacteria using phase contrast microscopy would not significantly widen the scope of this paper. We have added the following text to the discussion to clarify the conclusions we draw from our results (lines 448-452):

"The results described here demonstrate that the method is effective for both confocal laser scanning and brightfield microscopy, underscoring its versatility. Previously, synthetic images created with cycleGAN have also been used to train image analysis models for scanning transmission electron microscopy [18]. Together, these studies show that the approach can be effective for a variety of imaging modalities."
